# m^6^A: Widespread regulatory control in virus replication^☆^

**DOI:** 10.1016/j.bbagrm.2018.10.015

**Published:** 2019-03

**Authors:** Oliver Manners, Belinda Baquero-Perez, Adrian Whitehouse

**Affiliations:** a School of Molecular and Cellular Biology, Faculty of Biological Sciences, University of Leeds, Leeds LS2 9JT, United Kingdom; b Astbury Centre for Structural Molecular Biology, Faculty of Biological Sciences, University of Leeds, Leeds LS2 9JT, United Kingdom

**Keywords:** m^6^A, Viral replication, Epitranscriptomics, RNA modification, Post-transcriptional gene regulation, Virus-host interactions

## Abstract

N^6^-methyladenosine (m^6^A) is a highly pervasive and dynamic modification found on eukaryotic RNA. Despite the failure to comprehend the true regulatory potential of this epitranscriptomic mark for decades, our knowledge of m^6^A has rapidly expanded in recent years. The modification has now been functionally linked to all stages of mRNA metabolism and demonstrated to regulate a variety of biological processes. Furthermore, m^6^A has been identified on transcripts encoded by a wide range of viruses. Studies to investigate m^6^A function in viral-host interactions have highlighted distinct roles indicating widespread regulatory control over viral life cycles. As a result, unveiling the true influence of m^6^A modification could revolutionise our comprehension of the regulatory mechanisms controlling viral replication. This article is part of a Special Issue entitled: mRNA modifications in gene expression control edited by Dr. Soller Matthias and Dr. Fray Rupert.

## Highlights

• m6A methylation is functionally linked to all stages of mRNA metabolism and regulates a variety of biological processes.
• m^6^A methylation marks both the genomic RNA and mRNAs of multiple viruses.
• m6A methylation plays a significant role in modulating the replication of many viruses.

## 1 Introduction

**Fig. 1:**
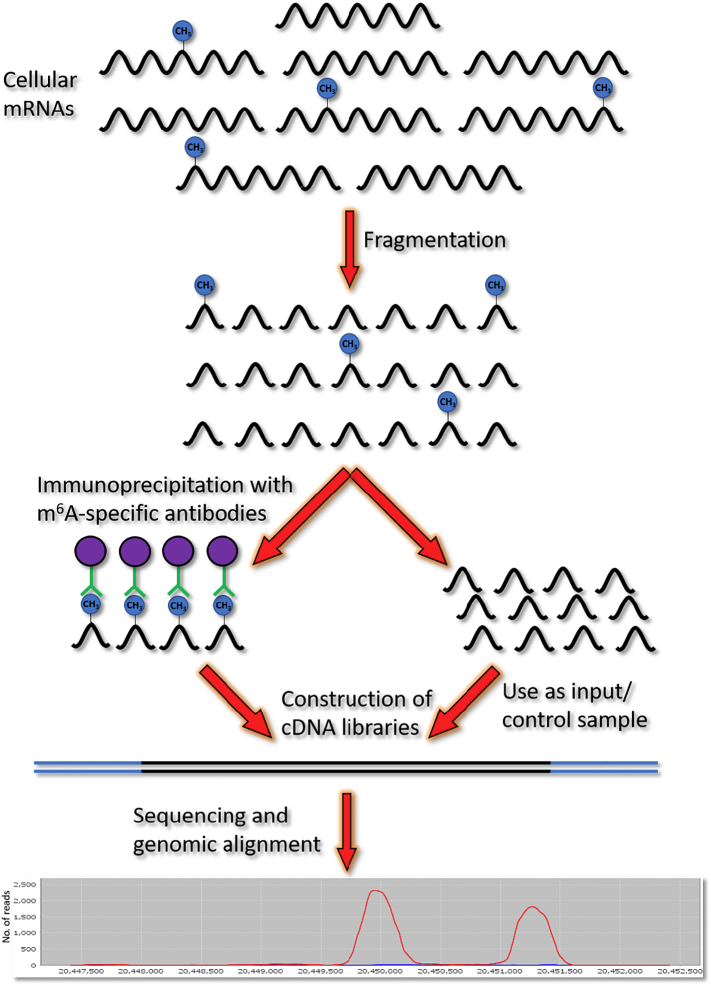
MeRIP-seq. The general procedure for MeRIP-seq involves the shearing of poly(A)^+^-selected mRNAs into 100–200 nt fragments followed by immunoprecipitation using m^6^A-specific antibodies attached to magnetic beads. The antibodies recognise N^6^-methylation but are therefore unable to distinguish m^6^A from the related RNA modification 2-*O*-dimethyladenosine (m^6^A_m_). Immunoprecipitated RNA fragments are reverse transcribed, used to construct cDNA libraries and subjected to deep sequencing. Reads are then mapped to specific transcripts and 100–200 nt peaks, containing sites of m^6^A methylation, are called using bioinformatic detection algorithms. A portion of the non-precipitated RNA is used as the input sample.

Although the internal modification of RNA residues in mammalian cells was first identified over 40 years ago, recent technological advances are only now beginning to unravel the functional importance of these changes in widespread physiological processes [1]. While over 100 distinct modifications comprise the epitranscriptome, most are constrained to noncoding RNAs such as tRNAs, rRNAs and snRNAs. However, the most prevalent internal modification of messenger RNAs (mRNAs), m^6^A, decorates the transcriptome to bring about profound changes in mRNA biology [[2], [3], [4]]. For several decades, m^6^A has been known to mark both the genomic RNA and mRNAs of multiple viruses although the precise functional importance of m^6^A in the life cycles of these viruses is still relatively unknown [[5], [6], [7], [8], [9], [10], [11]]. However, in the last few years, several publications have suggested this modification plays a significant and tantalising role in modulating viral replication.

m^6^A was originally identified on cellular mRNAs at a prevalence of approximately three modifications per transcript [1]. However, early technologies reliant on the quantification of m^6^A in RNA lysates could not map individual m^6^A sites to specific transcripts and were unable to determine the true variability of m^6^A content across cellular mRNAs. Consequently, the development of a novel methylated RNA immunoprecipitation-sequencing (MeRIP-seq or m^6^A-seq) method for mapping of the m^6^A methylome in 2012 was a huge breakthrough in the study of the epitranscriptome and reignited interest in RNA modifications (Fig. 1). In recent years, MeRIP-seq and subsequent enhanced versions of the technique have been harnessed to divulge crucial insight into the topology of m^6^A in the cellular transcriptome [12,13]. Some mRNAs, especially those of housekeeping genes, have been found to contain no m^6^A while others contain many sites of methylation. Furthermore, m^6^A is clustered in 3′ UTRs and near stop codons [14]. Together, these insights into the unequal distribution of m^6^A on cellular transcripts allude to fundamental regulatory roles for this post-transcriptional modification in mRNA biology.

## 2 The m^6^A machinery

### 2.1 Readers, writers and erasers

**Fig. 2:**
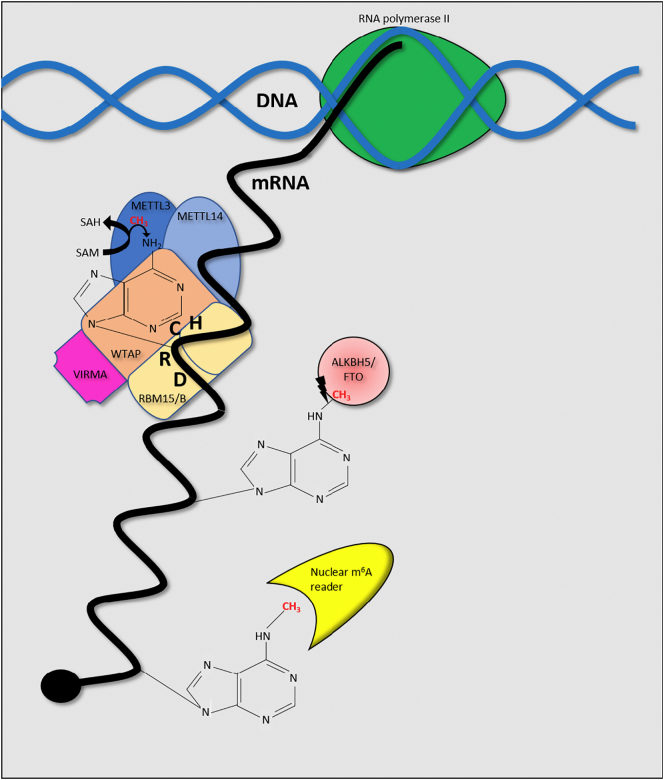
Dynamics of m6A. m^6^A is thought to be added to mRNAs at DRACH consensus sites. A methyl group is donated to the adenine base through the hydrolysis of S-adenosylmethionine to S-adenosylhomocysteine (SAH) by the methyltransferase complex, which contains a number of proteins crucial for efficient localisation and catalytic activity. Removal of the modification is undertaken by the m^6^A erasers ALKBH5 and FTO while recognition of m^6^A is carried out by m^6^A readers.

The reversible addition and removal of m^6^A upon cellular mRNAs is thought to be dynamically regulated by m^6^A writers and erasers allowing rapid adjustment of mRNA fate and thus regulatory control over various physiological processes (Fig. 2). However, the reversibility of m^6^A and wider RNA modifications is disputed by some groups which claim that significant and widespread demethylation does not occur in most cell types [15]. Similarly, although m^6^A has often been suggested to be added in a co-transcriptional manner, METTL3, METTL14 and ALKBH5 are all detectable among both nuclear and cytoplasmic cellular fractions [[16], [17], [18]]. Furthermore, m^6^A is found in the RNA of cytoplasmically-replicating viruses suggesting that post-transcriptional addition of m^6^A may also take place [18].

**Fig. 3:**
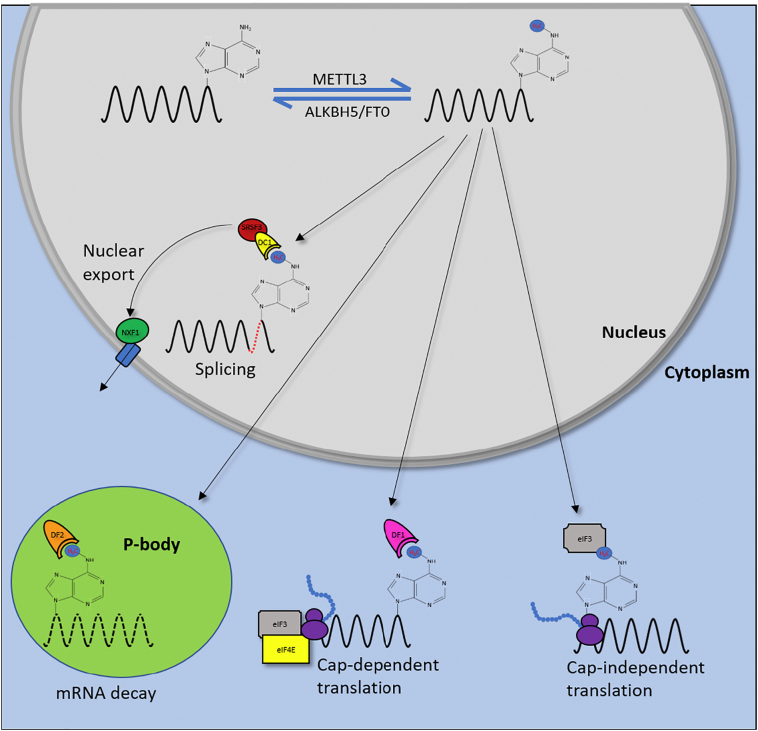
Biological functions of m6A. Following the dynamic m^6^A-modification of mRNAs in the nucleus through the actions of the methyltransferase complex and m^6^A erasers, the methylation site is bound by m^6^A readers such as DC1, DF1–3 and eIF3 in both the cytoplasm and nucleus. Depending on the context of the m^6^A residue within a transcript, the fate of the mRNA may be diverted towards splicing, export, translation or decay.

The deposition of m^6^A is catalysed by a large heteromultimeric methyltransferase complex or m^6^A writer complex, within which methyltransferase-like 3 (METTL3) is the catalytically active subunit and transfers a methyl group to adenosine residues through its S-adenosylmethionine (SAM) activity [[19], [20], [21]]. Using its adaptor protein methyltransferase-like 14 (METTL14), which adopts a structural role essential for recognition of the RNA substrate, METTL3 deposits m^6^A on cellular transcripts at the preferred DR(m^6^A)CH (D = A, G or U; R = A or G; H = A, C or U) consensus sequence [13,[22], [23], [24]]. Wilms Tumour 1 associated protein (WTAP) is responsible for the localisation of the METTL3-METTL14 heterodimer to nuclear speckles; whereas interaction partners RBM15 and RBM15B are proposed to regulate the selective distribution of m^6^A to only a proportion of total transcriptomic DRACH sites [25,26]. Finally, KIAA1429 is suspected to act as protein scaffold maintaining the structural integrity of the m^6^A methyltransferase complex, but it has also recently been suggested to mediate the preferential enrichment of m^6^A in 3′ UTRs and near stop codons [27,28]. The deletion of any of these components of the m^6^A writer complex leads to a profound loss of m^6^A methylation on cellular transcripts, emphasising the necessity for each subunit in efficient control of m^6^A dynamics [21,25,27,29]. However, the additional proteins ZC3H13 and HAKAI have recently been found to comprise the m^6^A methyltransferase complex indicating that additional factors regulate the activity and selectivity of m^6^A methylation [28,[30], [31], [32], [33]]. To date, two m^6^A erasers have been proposed to revert m^6^A to adenosine residues and facilitate the dynamicity of the modification, α-ketoglutarate-dependent dioxygenase alkB homolog 5 (ALKBH5) and fat mass obesity protein (FTO). Only subtle changes in m^6^A content have been observed in ALKBH5-depleted cells and knockout mice are mostly normal apart from impaired fertility; however, it is possible that the demethylase acts on only a fraction of specific m^6^A sites in specific sequence or structural contexts [34]. Although some evidence suggests FTO instead demethylates m^6^A_m_, a related RNA modification adjacent to the m^7^G 5′ cap structure on approximately 35% of cellular mRNAs, the extent to which the demethylase targets m^6^A remains unclear [35,36]. Nevertheless, the deletion of both proteins increases global m^6^A/_m_ content indicating these erasers contribute additional molecular fine-tuning to the regulation of mRNA biology [[34], [35], [36]].

m^6^A exerts its influence over mRNAs *in cis* by recruiting RNA binding proteins, known as m^6^A readers, which recognise the site of modification and direct the methylated transcript towards distinct biological fates (Fig. 3). The best characterised among this group are the YT521-B homology (YTH) domain containing proteins including YTHDF1 (DF1), YTHDF2 (DF2) and YTHDF3 (DF3), which reside in the cytoplasm, and YTHDC1 (DC1) which adopts a nuclear localisation [37,38]. The final YTH protein is YTHDC2, however this protein is poorly characterised, unrelated to the other members of its family and further work is required to determine whether DC2 targets m^6^A. The YTH RNA-binding motif contains an aromatic cage comprised of three tryptophan residues which can specifically bind to the methyl group through hydrophobic interactions [39]. m^6^A also reduces base pair stability and is found in regions with reduced RNA structure; though importantly, a recent study has demonstrated that m^6^A can stabilise regions of RNA under certain structural contexts [40]. It is suggested that m^6^A can permit RNA unfolding and improve the accessibility of certain RNA binding proteins to their target sites. As a result, proteins which exploit this ‘m^6^A switch’ mechanism such as HNRNPC and HNRNPG have also been suggested as m^6^A readers despite the indirect nature of their interaction [41,42]. However, recently a new type of m^6^A reader protein was described which utilises a common RNA binding motif, the KH domain, in cooperation with flanking regions to selectively bind methylated adenosines [43]. The amounting evidence that a myriad of m^6^A readers exist suggests that m^6^A has evolved as an integral cellular mechanism that permits widespread regulatory control over gene expression.

### 2.2 Functions of m^6^A

The life of an mRNA includes processing, nuclear export, translation and decay. The earliest evidence that m^6^A plays a regulatory role in this biological cycle arises during splicing. In one mechanism, the reduction in base pair stability associated with an m^6^A residue improves the accessibility of HNRNPC and HNRNPG to their respective U-rich and purine-rich binding sites, facilitating the alternative splicing of target mRNAs [41,42]. Furthermore, the depletion of a proposed m^6^A reader, HNRNPA2B1 has been suggested to phenocopy the effect of METTL3 depletion on the alternative splicing of certain primary microRNAs [44]. Recent studies indicate this protein also utilises an m^6^A switch mechanism, thus the m^6^A-dependent binding of HNRNPA2B1 to pre-mRNAs could similarly regulate their processing [45]. Finally, functional studies into DC1 have identified that the nuclear YTH protein facilitates the subcellular localisation of the pre-mRNA splicing factor SRSF3 to nuclear speckles; but repels SRSF10, leading to specific exon-inclusion patterns [46,47]. Furthermore, multiple bodies of evidence suggest DC1 suppresses the recognition of a splice site in the *Drosophila* Sxl transcript, through the binding of an m^6^A site, to control sex determination [[48], [49], [50]]. Finally, a recent report has demonstrated that the majority of m^6^A peaks upon newly transcribed mRNAs lie within introns and correlate with reduced splicing efficiency [16]. In addition, m^6^A sites were also enriched around 5′ splice junctions; therefore, through the deployment of its reader proteins, m^6^A influences the alternative splicing of thousands of exons.

Recent studies involving DC1 and the m^6^A writer complex have further expanded the known functions of m^6^A to involve the regulation of mRNA export. DC1 facilitates the RNA-binding of both the adaptor protein SRSF3 and the major mRNA export receptor NFX1, which in turn drives the nuclear export of the methylated transcript [47]. Accordingly, depletion of DC1 results in increased nuclear residence times of modified mRNAs, independent of splicing. Thus, m^6^A could act as a non-canonical nuclear export signal to be decoded by DC1, which in turn delivers the methylated transcript to NFX1.

Once in the cytoplasm, m^6^A residues have also been proposed to enhance the translational efficiency of certain transcripts using eIF4E cap-dependent or cap-independent mechanisms. In the former method, DF1 binds 3′ UTR m^6^A and interacts with the 5′ UTR-associated eukaryotic initiation factor 3 (eIF3) to promote translation; perhaps through the stabilisation of the 5′-3′ looping mechanism observed during canonical translation initiation [3]. Early research into DF1–3 analysed heterologously expressed DF proteins and identified distinct functions for each YTH m^6^A reader. However, DF1–3 show nearly identical overlap with m^6^A sites and recent studies examining endogenous DF proteins suggest these m^6^A readers all promote translation [21,51,52]. Further study is required to determine whether the DF proteins behave redundantly. While the YTH proteins can promote translation through association with 3′ UTR-m^6^A, m^6^A-crosslinking assays have also shown that eIF3 is able to directly bind m^6^A in the 5′ UTRs of cellular transcripts using a multi-domain interface [4]. This leads to recruitment of the 43S pre-initiation complex, independent of the eIF4E cap-binding protein, promoting a unique, non-canonical form of m^6^A-driven translation initiation. This surrogate mechanism may be particularly important under cellular stress where eIF4E activity is hindered. Reinforcing this hypothesis, m^6^A increases at the 5′ UTRs of cellular transcripts in response to heat shock suggesting this modification may be used to bypass the dependency on a cap binding protein in the translation of mRNAs [53].

An early study into the function of the YTH proteins found that DF2 directs methylated-transcripts towards RNA decay. Accordingly, in cells where DF2 is depleted, its targets showed elevated half-lives [2]. The protein is proposed to bind m^6^A through its C-terminal YTH domain and then relocalise methylated transcripts to P-bodies for degradation through interactions at its N-terminal low-complexity region. However, given that mass spectrometry has shown that none of the DF proteins are enriched in P-bodies this interaction may be transient [2,54]. Additional research has provided further insight into this process whereby, prior to translocation of m^6^A-methylated transcript, mRNAs are deadenylated through interactions between DF2 and members of the CCR4-NOT deadenylase complex [55]. Importantly, this study found that all DF proteins interact with the CCR4-NOT complex reinforcing evidence that these m^6^A-readers behave redundantly [18,52].

## 3 m^6^A in viral infections

Although there is much ground to cover in the elucidation of m^6^A function, major developments in the field of epitranscriptomics now permit the study of RNA modification during viral life cycles. Currently, only limited evidence has been gathered in viruses (Table 1). Most of these reports have involved the depletion of the m^6^A machinery followed by assessment of any associated changes in viral replication. However, these changes could be indirect due to alterations in the fate of cellular RNAs rather than viral transcripts. As a result, some groups have specifically mutated sites of m^6^A in viral transcripts to elucidate the function of the modification at certain loci. Nevertheless, all studies into the function of m^6^A in viral life cycles suggest epitranscriptomics has the potential to profoundly change our understanding of virus-host interactions.

**Table 1:** List of viruses in which m^6^A has been functionally investigated through depletion or overexpression of components of the m^6^A machinery.

Virus	Phenotype of writer depletion	Phenotype of eraser depletion	Phenotype of reader depletion	Phenotype of reader overexpression	Specific function of m^6^A	Reference
HIV-1	Antiviral (METTL3; METTL14)	Proviral (ALKBH5)	–	–	Nuclear export	[56]
	–	–	Antiviral (DF2)	Proviral (DF1–3)	mRNA abundance	[51]
	Antiviral (METTL3; METTL14)	Proviral (FTO; ALKBH5)	Proviral (DF1–3)	Antiviral (DF1–3)	Reverse transcription	[57]
HCV	Proviral (METTL3; METTL14)	Antiviral (FTO)	Proviral (DF1–3)	–	Virion packaging	[58]
ZIKV	Proviral (METTL3; METTL14)	Antiviral (FTO; ALKBH5)	Proviral (DF1–3)	Antiviral (DF1–3)	–	[18]
IAV	Antiviral (METTL3)	–	–	Proviral (DF2)	mRNA abundance	[59]
KSHV	Antiviral^a^ (METTL3)	Proviral^a^ (FTO)	–	–	ORF50 pre-mRNA splicing	[60]
	–	–	Proviral^b^ (DF2)	Antiviral^b^ (DF2)	–	[61]
	Proviral^a^ and antiviral^b^ (METTL3)	–	Proviral^a^ and antiviral^b^ (DF2)	–	–	[62]
SV40	Antiviral (METTL3)	–	Antiviral (DF2)	Proviral (DF2, DF3)	Nuclear export, Translation	[63]
HBV	Proviral and antiviral (METTL3 & METTL14)	Proviral and antiviral (ALKBH5; FTO)	Proviral and antiviral (DF2, DF3)	–	mRNA abundance, reverse transcription	[64]
AMV	–	Antiviral (ALKBH9B)	–	–	Interaction with viral coat protein	[65]

a B-cell line.

b Endothelial cell line.

### 3.1 m^6^A in HIV-1 infection

During HIV-1 replication, viral mRNAs are subjected to both cap-dependent and independent forms of translation, a non-canonical form of nuclear export reliant on the HIV-1 protein Rev and extensive alternative splicing [[66], [67], [68]]. Given the known functions of m^6^A in the control of these processes, it is conceivable that the modification plays crucial roles in the epitranscriptomic regulation of HIV-1 gene expression.

In 2016, three studies were conducted investigating the role of m^6^A in HIV-1 infection; all using MeRIP-seq or the enhanced m^6^A mapping technology PA- m^6^A-seq to identify specific sites of m^6^A methylation on HIV-1 RNA [51,56,57]. Furthermore, two of these studies performed cross-linking immunoprecipitation (CLIP) assays to examine the binding sites of the DF proteins [51,57]. Lichinchi and colleagues identified 14 distinct methylation peaks in the 5′ and 3′ UTRs, coding sequences and splicing regulatory sequences, suggesting a variety of functions for m^6^A in HIV-1 genomic RNA (gRNA). All three studies reported shared 3′ UTR m^6^A clusters in the 3′ 1.4 kb of the 9.2 kb HIV-1 RNA genome. However, Kennedy and colleagues reported between zero and two further 3′ UTR m^6^A clusters in three HIV-1 isolates while Tirumuru et al. only identified one additional 5′ UTR m^6^A peak. Much of the variation between these studies likely arises from differences in bioinformatic methods of calling m^6^A peaks or alternatively due to the use of different HIV-1 isolates in varying cell types. In the former case, implementation of a consistent and effective method for identifying m^6^A sites, of which many are being developed, will dissolve these mapping incongruities in future studies [69]. Nevertheless, all three studies agree on the presence of m^6^A at the 3′ end of HIV-1 gRNA demonstrating indisputably the epitranscriptomic modification of the HIV-1 RNA genome.

To address the role of m^6^A in HIV-1 infection, two of the studies modified expression levels of m^6^A writers, readers and erasers in order to observe any associated changes in viral replication efficiency. Lichinchi et al. carried out shRNA-mediate depletion of METTL3, METTL14 and ALKBH5, then quantified RNA levels of the HIV-1 GP120 envelope glycoprotein and immunoblotted for the viral capsid protein p24, 72 h post-infection [56]. Knockdown of METTL3 and METTL14 decreased GP120 and p24 levels and accordingly an additive effect was identified for depletion of both writers. Conversely, a prominent increase in GP120 and p24 was seen in ALKBH5-depleted cells. In agreement with these results, Tirumuru and colleagues also depleted METTL3, METTL14, FTO and ALKBH5 finding a decrease in structural polyprotein precursor p55 Gag and p24 protein levels associated with knockdown of components of the m^6^A writer complex and the opposite effect for depletion of the demethylases [57]. Together, these studies suggest m^6^A positively regulates HIV-1 replication.

Harnessing an alternative approach to interrogate m^6^A function during HIV-1 infection, Kennedy and colleagues overexpressed the reader proteins DF1–3. They observed enhanced expression of the HIV-1 mRNAs Nef, Tat and Rev in addition to increased protein levels of p55 Gag, p24 and Nef [51]. In contrast, CRISPR-Cas-mediated deletion of *YTHDF2* in HIV-1-infected CD4^+^ T cells was associated with a significant decline in p24 and Nef protein levels, supporting the hypothesis that m^6^A positively regulates HIV-1 replication. However, Tirumuru et al. observed contradictory results associated with modulation of DF1–3 expression. Overexpression of these m^6^A readers in HeLa cells inhibited HIV-1 infection by 10-fold and led to substantial downregulation of Gag protein; while a 4–14-fold increase in HIV-1 infectivity was observed following DF1–3 depletion [57]. These observations were corroborated in a CD4^+^ T-cell line and primary CD4^+^ T-cells. Further examination found that overexpression of DF1–3 led to a decrease in late reverse transcription products while their depletion reversed this effect, suggesting that m^6^A inhibits HIV-1 reverse transcription. In turn, this increase or decrease in reverse transcription products was positively correlated with changes in the expression gag mRNA and therefore HIV-1 gene expression. The authors of Kennedy et al. have since suggested the use of a modified HIV-1 strain by the Tirumuru and colleagues, containing a firefly luciferase HIV-1 reporter to measure viral infection, as a possible source for the divergent results. They suggest that pronounced sites of m^6^A modification in firefly luciferase mRNA may affect how the DF proteins influence HIV-1 replication [70]. Nevertheless, more recent evidence from Tirumuru and colleagues using wild type virus suggests their previous observations were not affected by methylation of firefly luciferase RNA. Furthermore, they identified that DF1–3 bind preferentially to two 5′ UTR sites in HIV-1 gRNA to reduce levels of viral gRNA and both early and late reverse transcription (RT) products [71]. In addition, the authors demonstrate an RNA-dependent interaction between DF1–3 and HIV-1 Gag but not p24. Thus, despite some differences, these studies demonstrate unequivocally that the m^6^A machinery plays profound roles in HIV-1 replication.

To assess the precise function of four m^6^A peaks mapped to the 3′ 1.4 kb of the HIV-1 NL4–3 genome, Kennedy and colleagues transfected HEK 293T cells with two *Renilla* Luciferase (RLuc)-based indicator plasmids containing either two or four of these putative m^6^A clusters [51]. Interestingly, these putative m^6^A clusters in the 3′ 1.4 kb of the HIV-1 genome mostly localised to the 3′ UTR in their corresponding viral mRNAs. The two plasmids were transfected in either wild type or mutant form where all DRACH consensus sites within the putative m^6^A regions were mutated to prevent methylation. Both plasmids containing wild type HIV-1 sequences expressed significantly higher RLuc mRNA and protein compared to their respective m^6^A-deficient forms. However, the level of enhancement was equivalent at both RNA and protein levels, suggesting that 3′ UTR m^6^A increases the steady state RNA levels of HIV-1 transcripts without influencing translation. In addition, artificial tethering of DF1–3 to the 3′ UTR of an RLuc indicator plasmid phenocopied this effect by increasing RLuc expression. Together, these results suggest that the DF proteins bind m^6^A residues within the 3′ UTR of HIV-1 transcripts and enhance expression of viral mRNAs *in cis*.

To tether a specific functional role to an m^6^A site discovered through MeRIP-seq, Lichinchi and colleagues examined an m^6^A peak which localised to stem loop IIB of the Rev response element (RRE). Binding of Rev protein to its RRE facilitates the nuclear export of viral mRNAs and is therefore a pivotal step in HIV-1 replication. Using m^6^A-sensitive and insensitive primers, the presence of two m^6^A sites at nucleotides 7877 and 7883 was confirmed within this region [56]. To identify whether the m^6^A-modification of these sites affects the affinity of Rev for its response element, Lichinchi and colleagues mutated these residues to prevent methylation. No significant effect on viral replication or nuclear export was associated with mutation of A7877. However, a striking decrease in both viral replication and RNA export was observed when m^6^A was abrogated at A7883. Furthermore, comparison of this position in 2501 HIV-isolates identified a mutation rate of just 0.28%; far lower than the frequencies associated with other adenosine nucleotides in stem loop IIB. Previous *in vitro* structural studies using NMR have demonstrated that A7883 bulges out of the stem region of the RRE and associates with Rev at position W45 [72]. However, depletion of METTL3 and METTL14 also reduced nuclear export of viral RNAs while ALKBH5 knockdown produced the opposite effect. Taken together, these results not only suggest that residue A7883 is critical for the Rev-RRE interaction and nuclear export of HIV-1 RNA, but additionally that this adenosine nucleotide must be m^6^A-modified. As a result, Lichinchi and colleagues provide a compelling example of the importance of m^6^A in viral-host interactions. Importantly however, neither Kennedy et al. nor Tirumuru et al. identified this RRE-located m^6^A peak in their MeRIP-seq data sets. As a result, further epitranscriptomic characterisation of HIV-1 gRNA is needed to fully understand the regulatory influence of m^6^A in HIV-1 replication.

### 3.2 m^6^A in flaviviruses

The investigation of m^6^A in HIV-1 infection was followed by two publications in late 2016, providing unexpected evidence of m^6^A in the positive sense, single-stranded RNA genomes of cytoplasmically-replicating flaviviruses. Although the m^6^A methyltransferase complex and ALKBH5 have been previously described as confined to the nucleus, both studies immunoblotted against METTL3, METTL14 and ALKBH5 in both nuclear and cytoplasmic fractions of mock and flavivirus-infected cells. They identified all three proteins in both fractions, suggesting m^6^A writers and erasers can enter the cytoplasm where they facilitate the methylation and demethylation of flaviviral RNAs [18,58].

Gokhale and colleagues carried out MeRIP-seq to map the m^6^A landscape in Hepatitis C virus (HCV), identifying 19 peaks in the total 9.6 kb RNA genome. PAR-CLIP mapping of FLAG-tagged DF1–3 identified 42 binding sites; only 50% of which overlapped with regions of m^6^A reported by MeRIP-seq [58]. Although other studies have indicated that the YTHDF proteins bind almost all m^6^A sites, this discrepancy can be partially attributed to the binding of these proteins to non-methylated target sites [38]. To identify any conservation in the m^6^A landscape between flaviviruses, Gokhale and colleagues performed MeRIP-seq on the RNA genomes of Dengue, yellow fever, West Nile and two isolates of Zika virus (ZIKV). Interestingly, a fraction of the mapped m^6^A sites localised to similar regions among all the viruses, including the NS3 and NS5 genes, which may suggest a conserved role for m^6^A in their post-transcriptional regulation. Lichinchi et al. identified 12 m^6^A peaks in the full length 10.8 kb ZIKV RNA genome through MeRIP-seq [18]. Comparison of these putative m^6^A sites with four additional ZIKV strains demonstrated a high degree of sequence similarity indicating the m^6^A landscape is conserved in this virus. Furthermore, the identification of these m^6^A clusters in the NS3 and NS5 genes in both studies demonstrates conclusively the m^6^A-modification of flaviviral RNA.

In a similar method of investigation to those carried out in the HIV-1 studies, both Lichinchi et al. and Gokhale et al. depleted the cellular m^6^A machinery and screened for associated changes during flaviviral infection. Depletion of METTL3 and METTL14 by Gokhale and colleagues increased extracellular HCV RNA levels and infectious virion production, whereas knockdown of FTO had the opposite effect and reduction of ALKBH5 expression did not affect viral titre [58]. However, the use of a *Gaussia*-luciferase reporter virus to assess HCV RNA replication found no significant change in luciferase levels upon depletion of the m^6^A machinery, suggesting that m^6^A instead restricts the production or release of infectious virions. In agreement with these results, shRNA-mediated knockdown of METTL3 and METTL14 in ZIKV-infected HEK293T cells by Lichinchi et al. increased viral titre and ZIKV RNA levels, but also enhanced the expression of ZIKV envelope protein [18]. In contrast however, depletion of ALKBH5 or FTO decreased viral titre, ZIKV RNA expression and levels of envelope protein. In both studies, these results were validated by overexpressing these components of the m^6^A machinery and observing the reverse effects to those seen for depletion. Excluding ALKBH5 knockdown in HCV-infected cells, the results of the two papers demonstrate the negative regulation of flavivirus life cycles by the m^6^A landscape. It remains unclear why these viruses would retain m^6^A if it negatively impacted their life cycles given that consensus sites could be quickly lost through selection. Perhaps m^6^A positively regulates these viruses at certain stages of their replication or, as suggested in both reports, the modification facilitates escape from host antiviral immune responses. Indeed, the m^6^A modification of several *in vitro* synthesised RNAs suppresses recognition by the host pattern recognition receptors, TLR3, TLR7, TLR8 and RIG-1 [73,74].

Next, DF1–3 were depleted to identify whether these readers mediate the negative regulation of ZIKV and HCV RNA by m^6^A. In both cases, knockdown of DF1–3 increased levels of extracellular viral RNA [18]. Furthermore, in ZIKV-infected cells, these observations were corroborated by DF1–3 overexpression which reduced extracellular viral RNA levels. The studies also demonstrated the discriminatory binding of YTH proteins to HCV and ZIKV RNA by immunoprecipitation of FLAG-tagged DF proteins followed by qRT-PCR. Finally, Gokhale and colleagues demonstrated the redistribution of all three DF proteins to cytoplasmic sites of HCV virion assembly, known as lipid droplets. Taken together, these results demonstrate that the modulation of RNA levels in HCV and ZIKV is functionally linked to DF binding of m^6^A-methylated viral RNA.

To interpret the functional relevance of a specific m^6^A cluster in the HCV RNA genome, Gokhale and others selected one region in the E1 gene which they had identified as bound by all three DF proteins and m^6^A-modified through their previous mapping experiments. Within this location, a cluster of four potential m^6^A sites were mutated to abolish the potential for N^6^-methylation without affecting the encoded amino acid sequence [58]. Electroporation of m^6^A-deficient HCV RNA into Huh7 cells resulted in three-fold higher HCV virion production compared with control HCV RNA. Furthermore, it was demonstrated that E1-mutated HCV RNA was bound more efficiently by HCV core protein, enhancing its packaging into nascent virions. Thus, Gokhale et al. demonstrated a specific mechanism for m^6^A-mediated regulation of HCV viral particle production.

### 3.3 m^6^A and influenza A

Influenza A virus (IAV) contains a segmented, negative sense, single-stranded RNA genome and replicates in the nucleus. Several decades ago, the presence of approximately 24 m^6^A-modified residues were identified on IAV mRNAs with eight sites concentrated onto the haemagglutinin (HA) mRNA segment; encoding a major viral envelope protein [11,75]. However, at the time, these sites of modification could not be accurately mapped and thus m^6^A function could not be elucidated. In a recent study, Courtney et al. mapped the topology of m^6^A on both the positive-cRNA and negative-vRNA segments of the IAV genome using PA-m^6^A-seq and PAR-CLIP for DF1–3. With some exceptions, the m^6^A peaks and DF binding sites were consistent [59]. The results identified an abundance of m^6^A in the genes encoding highly expressed structural proteins but far fewer sites of modification in mRNAs encoding the RNA polymerase subunits.

Utilising the same interrogatory methods employed for other viruses, Courtney and colleagues abrogated METTL3 expression through CRISPR/Cas-mediated knockout in the human lung epithelial cell line A549 [59]. Subsequent measurement of the IAV replicative ability in these METTL3-deficient cells identified an eight-fold decrease in the expression of viral structural proteins including NS1, NP and M2 compared to wild type virus. In addition, reduced viral titre and mRNA levels of NP and M2 were also reported in the METTL3 mutants. Supporting these results, overexpression of DF2 enhanced the expression of the same viral proteins and mRNAs, in addition to increasing viral titre roughly 5-fold. Surprisingly however, no significant effect could be detected for DF1 or DF3 overexpression, suggesting these m^6^A readers may not play a substantial role during IAV infection, at least in A549 cells. Nevertheless, together these data indicate that the m^6^A-modification of IAV RNA positively regulates the replication of the virus.

In agreement with previous studies, Courtney and others identified eight m^6^A sites in the HA cRNA segment and a further nine peaks in HA vRNA. To identify any regulatory importance for these m^6^A residues, Courtney and colleagues produced two IAV HA mutant viruses; each containing either a vRNA or cRNA segment in which the majority of m^6^A sites were silently mutated to prevent N^6^-methylation without affecting the amino acid sequence [59]. In cells infected with m^6^A-deficient virions, HA was specifically downregulated at both the protein and mRNA level without any effect on expression of other viral genes including NS1 and M2. Furthermore, these HA mutants displayed significantly attenuated pathogenicity in infected mice compared to their parental wild type highly pathogenic IAV strain. Thus, m^6^A plays a crucial role in modulating expression of HA and therefore IAV infectivity.

Employing a strategy absent from previous studies in viruses, Courtney et al. attempted to interpret how m^6^A positively regulates IAV by comparing the immune response to the m^6^A-depleted HA mutants and wild type virus. However, no difference was detected in the expression of various anti-viral innate immune response proteins including RIG-1, MGA5 and Interferon β suggesting m^6^A positively regulates IAV RNA levels through mechanisms other than downregulation of immune activity.

Given that the m^6^A enhances the expression of both splice variants of the NS1 and M2 genes at equal ratios and similarly increases mRNA and protein levels of IAV viral genes at identical proportions, Courtney and colleagues suggest the effects mediated by m^6^A do not affect splicing or translation [59]. Furthermore, m^6^A residues enhance the abundance of both IAV HA mRNA and vRNA; the latter of which is constrained to the nucleus until late in the viral life cycle where it is packaged into virions. Given this information, Courtney et al. suggest an m^6^A-mediated effect on RNA export is unlikely and instead the modification increases IAV RNA abundance through enhanced stability or replication.

### 3.4 m^6^A and KSHV

Kaposi's sarcoma-associated virus (KSHV) is a double stranded DNA virus associated with the endothelial tumour Kaposi's sarcoma and two lymphoproliferative disorders [76,77]. Like all herpesviruses, KSHV undergoes distinct latent and lytic life cycles. In the latent stage, KSHV is episomally maintained in the host nucleus and expresses only a few genes to sustain a state of dormancy. Upon reactivation from the latent phase, expression of ORF50, encoding the master regulator of lytic replication RTA, is sufficient to initiate a temporally regulated cascade of gene expression leading to the production of infectious virions [78].

To date, three independent studies have been published detailing the m^6^A-modification of both lytic and latent KSHV transcripts. Ye and colleagues conducted MeRIP followed by qPCR of viral transcripts to demonstrate the extensive m^6^A modification of the KSHV genome [60]. The abundance of lytic transcripts and their m^6^A content increased robustly following induction of various KSHV-infected cell lines with multiple different stimuli. Additionally, Tan and others identified numerous changes in the viral m^6^A landscape upon both infection of five cell lines with KSHV and following induction of lytic replication in two of these cell lines [61]. In all five latently-infected cell types, they found conserved m^6^A peaks in latent transcripts including LANA, vFLIP and vCyclin. Interestingly, these transcripts gained additional peaks when lytic replication was stimulated in the endothelial KiSLK and B-cell-derived TREX-BCBL1 cell lines. Furthermore, the studies identified numerous conserved and some cell type specific m^6^A peaks on viral lytic mRNAs following induction. Finally, Hesser and colleagues reported a 3-fold increase in cellular m^6^A content upon induction of iSLK.219 cells by liquid chromatography-tandem mass spectrometry [62]. They later attribute this change to the m^6^A-modification of the KSHV non-coding RNA PAN which is suggested to comprise more than 80% of nuclear polyA+ RNA levels during lytic reactivation [79,80]. In addition, MeRIP-seq was carried out to demonstrate that approximately one third of KSHV mRNAs become m^6^A methylated upon KSHV induction. Taken together, these studies suggest m^6^A might play a crucial role in both the establishment of KSHV infection and controlling the regulatory switch between lytic and latent KSHV replication programmes.

To assess whether modulation of m^6^A levels would affect KSHV lytic replication, Ye and colleagues performed lentiviral knockdowns of METTL3 and FTO in the KSHV-infected TREX-BCBL1 cell line and stimulated lytic gene expression [60]. METTL3-depletion reduced virion production and decreased both the mRNA and proteins levels of ORF50 and the early gene ORF57. Furthermore, addition of the drug 3-deazaadenosine (DAA), which inhibits SAM activity and thus the addition of m^6^A to RNA, abolished KSHV lytic replication. In contrast, FTO-depletion or addition of the FTO inhibitor meclofenamic acid (MA) enhanced the production of KSHV viral particles and expression of ORF50 and ORF57 [81]. Although these results suggest m^6^A positively regulates the production of KSHV virions, Tan and colleagues found that depletion of DF2 in KiSLK cells led to a four-fold increase in virion production alongside a two- to six-fold rise in expression of the viral mRNAs ORF50, ORF57, ORFK8 and ORF65 leading to concomitant increases in their protein levels while DF2 overexpression reversed these effects [61]. However, no consistent or significant effect was observed for overexpression of other YTH proteins which may be due to their lower expression levels relative to DF2. Tan and colleagues also showed that depletion of DF2 elevated the half-lives of lytic KSHV transcripts through actinomycin D treatment and confirmed a 1.5-fold increase in the half lives of LANA, ORF57, ORF59, ORFK8 and ORF65 by RT-qPCR. As a result, the authors suggest YTHDF2 may act as an antiviral cellular restriction factor by targeting KSHV transcripts for degradation in P-bodies or to proteins with decapping, deadenylation or exonuclease activity in a P-body independent mechanism.

Hesser and colleagues repeated the depletions of both METTL3 and the YTHDF proteins in both the iSLK.219 and TREX-BCBL1 cell lines and carried out a range of assays to determine the phenotypic effect on KSHV lytic replication [62]. Viral transfer assays, assessing the ability of GFP-expressing virions produced in endothelial cells to reinfect 293T cells, demonstrated that depletion of DF2 and METTL3 strikingly decreased infectious virion production. Additionally, while significant reductions in the abundance of the late viral transcript ORFK8.1 were only observed for knockdown of METTL3, depletion of DF2 reduced the levels of the immediate early, delayed early and late KSHV mRNAs ORF50, ORF37 and ORFK8.1 and also the RTA and ORF59 proteins. These observations may be the result of upstream alterations in the expression of early viral transcripts which in turn cause a reduction in the levels of mRNAs expressed later during KSHV reactivation. In agreement with Tan and colleagues, no significant or consistent effect could be observed for depletion of DF1 or DF3. Surprisingly however, when Hesser and colleagues repeated these assays in TREX-BCBL1 cells, METTL3 and DF2 depletion had no significant effect on infectious virion production nor ORF50 and ORF59 mRNA levels, but increased protein levels of RTA and ORF59 indicating that m^6^A restricts KSHV replication in these cells. As a result, the authors suggest that m^6^A could elicit both pro- and antiviral control over KSHV lytic replication depending on the host cell type. Conceivably, the differential methylation of DRACH sites, alternative recognition by m^6^A readers or the availability of certain host cell factors could contribute to the opposing functions of m^6^A observed in these cells. However, the observations of Hesser and colleagues are not fully consistent with those seen by the other two studies in the iSLK and TREX-BCBL1 cell lines. Consequently, the role of m^6^A in KSHV infection remains uncertain and future studies are required to resolve the outstanding discrepancies.

Given that m^6^A-abolition impaired the induction of the lytic transactivator RTA in TREX-BCBL1 cells and m^6^A has been functionally linked to splicing through DC1 recognition, Ye and colleagues investigated whether m^6^A might affect pre-mRNA splicing of ORF50 [46,60]. Induction of KSHV lytic replication increased both pre-mRNA and mRNA levels of ORF50, but in the presence of DAA, mRNA abundance significantly declined without significantly altering pre-mRNA levels. To show that m^6^A was important for this decrease in mRNA to pre-mRNA ratio, Ye and colleagues carried out MeRIP-seq to determine the m^6^A landscape of ORF50 mRNA, identifying 14 sites of methylation. Next, the ORF50 gene was cloned into a pCMV-myc plasmid and *in vitro* mutagenesis of the 14 methylation sites carried out to produce individual plasmids lacking a single ORF50 m^6^A site. The plasmids were transfected into HEK 293T cells to assess the effects on pre-mRNA splicing. Four of these plasmids, three of which lacked m^6^A in the single ORF50 intron and one of which was m^6^A-deficient at a site in exon 2, displayed significantly reduced ratios of mRNA to pre-mRNA suggesting that splicing had been impaired. Furthermore, coimmunoprecipitation and RNA immunoprecipitation experiments demonstrated that the m^6^A reader YTHDC1, SRSF3 and SRFS10 interact with each other and bind ORF50 mRNA. Finally, Ye and colleagues show that at these four site of modification within the ORF50 transcript, m^6^A regulates the association of the splicing factors SRSF3 and SRSF10 to control both exon inclusion and intron exclusion. Taken together, these results suggest m^6^A modulates alternative splicing within ORF50 pre-mRNA and thus regulates KSHV lytic replication.

### 3.5 m^6^A in SV40

Another dsDNA virus whose transcripts are subjected to m^6^A-methylation is SV40; a member of the polyomavirus family. Although the modification of SV40 mRNAs was elucidated several decades ago, the functional link between m^6^A methylation and viral replication has only recently been examined [5]. Tsai and others commenced by modifying expression of the m^6^A machinery to identify any associated changes in viral replication [63]. Overexpression of DF2 elevated expression of both the early large T antigen protein and the late structural protein VP1, while an increase in both the size and number of viral plaques were observed in viral plaque assays. Furthermore, a similar, but less profound effect was observed on overexpression of DF3. In contrast, CRISPR-Cas-mediated deletion of both DF2 and METTL3 reversed the effects seen for DF2 overexpression. Accordingly, these results suggest m^6^A positively regulates SV40 replication.

Consistent with strategies employed for previous viruses, Tsai and colleagues proceeded to map sites of m^6^A-modification within the SV40 genome through both PA-m^6^A-seq and PAR-CLIP for binding of DF2 and DF3 [63]. Although the binding sites identified through the two techniques were not identical, they were mostly overlapping; permitting the discovery of 13 m^6^A peaks within the SV40 genome including 11 in late region encoding structural proteins. Notably, nine of these peaks were detected in the VP1 gene which also forms the 3′ UTR of the transcripts VP2 and VP3.

To determine whether the enhancement of SV40 replication associated with modulating the m^6^A machinery resulted from changes to cellular or viral transcripts, Tsai and colleagues produced a hypomethylated SV40 mutant termed ‘VPm’ in which all 20 DRACH consensus sequences within the 11 putative m^6^A regions of late transcripts were disrupted [63]. PA-m^6^A-seq was used to demonstrate the complete abrogation of m^6^A at three locations and partial removal at a further six m^6^A sites in the VPm mutant. Next, Tsai et al. compared the replicative ability of VPm with wild type SV40 in three permissive cell lines; BSC40, CV-1 and Vero. A significant decrease in the expression of viral early and late proteins was observed, in combination with reduced plaque size, confirming that a reduction in m^6^A content on viral transcripts is responsible for impaired SV40 replication.

Given that SV40 undergoes a complex pattern of splicing and the nuclear reader DC1 has been reported to undertake m^6^A-directed splicing of cellular transcripts, Tsai and colleagues investigated whether VPm produced aberrant splicing patterns in late mRNAs through the RT-PCR of late transcripts. However, no significant difference in the expression pattern of SV40 transcript variants could be observed between cells infected by wild type and mutant viruses suggesting that m^6^A is not required for splicing of SV40 mRNAs.

To examine a specific role for m^6^A in the expression of late SV40 proteins, Tsai et al. produced constructs containing the VP1 gene derived from wild-type SV40 or VPm. When both constructs were transfected into HEK 293T cells, 10-fold lower VP1 protein levels were observed in cells expressing the hypomethylated VP1 transcript despite the lack of a significant change in mRNA abundance. However, comparing both cytosolic and nuclear fractions, a 2-fold decrease was identified in the cytosolic levels of the hypomethylated VP1 transcript suggesting m^6^A is important for the nuclear export of VP1 mRNA [63]. However, since this change cannot individually account for the 10-fold lower VP1 protein levels identified previously, Tsai et al. hypothesize that m^6^A primarily influences VP1 expression by enhancing its translation. Together, these results suggest m^6^A positively regulates SV40 replication through enhanced translation and export of viral late transcripts; providing yet another example of the regulatory importance of m^6^A in the life cycle of a virus. Supporting this conclusion, the addition of DAA profoundly reduces expression of SV40 late viral proteins; attesting to the potential for m^6^A as a molecular target in the treatment of viral infection [63].

### 3.6 m^6^A in HBV

Hepatitis B Virus (HBV) is a dsDNA virus which replicates through the reverse transcription of an RNA intermediate known as pregenomic RNA (pgRNA). The majority of HBV pgRNA is translated into the viral protein while the remainder is encapsidated with core and pol subunits, followed by reverse transcription to produce mature capsids. A recent study described dual-functionality for m^6^A in the HBV life cycle [64]. Initially, Imam and colleagues demonstrated the m^6^A-modification of HBV RNA by performing meRIP followed by qRT-PCR of methylated RNA using primers specific to a 3′ UTR sequence shared by all HBV transcripts. Importantly, viral RNA was methylated in two HBV-infected cell lines and the liver tissues of patients with chronic hepatitis B. Furthermore, the pool of pgRNA which is destined for reverse transcription was also shown to be methylated by meRIP-RT-qPCR following isolation from core particles. Finally, pgRNA was enriched in DF2 and DF3 immunoprecipitates following transfection of HBV-infected HepAD38 cells with FLAG-tagged DF2 and DF3 providing further evidence of m^6^A-modified HBV RNA.

To determine whether m^6^A exerts any effect on the HBV life cycle, Imam et al. simultaneously depleted the methyltransferase components METTL3 and METTL14, in addition to the independent knockdown of the m^6^A erasers FTO and ALKBH5 [64]. The results showed that METTL3 and METTL14-depletion increased expression of the viral proteins HBs and Hbc, while the reverse effect was observed in cells lacking FTO or ALKBH5 expression. Furthermore, knockdown of the m^6^A readers DF2 and DF3 recapitulated the increase in viral protein expression observed for depletion of METTL3 and METTL14, suggesting that m^6^A negatively regulates expression of HBV proteins. Interestingly, an increase in expression of the pgRNA was also seen for DF2- and DF3-knockdown; suggesting that the decrease in HBV protein levels is due to diminished RNA abundance rather than reduced translation. Confirming this hypothesis, the group measured the stability of HBV transcripts by actinomycin D treatment in cells lacking METTL3 and METTL14 or DF2. They observed over a two-fold increase in the half-life of pgRNA following depletion of these m^6^A machinery components suggesting that m^6^A negatively affects the stability of HBV RNA. Interestingly however, the group also measured the effect of m^6^A on the reverse transcription of HBV pgRNA by measuring core-associated DNA levels in cells lacking METTL3 and METTL14 or FTO. Reverse transcription was significantly reduced in cells lacking METTL3 and METTL14, but enhanced in those lacking FTO. Taken together, these results suggest a dual-role for m^6^A in the HBV life cycle involving the destabilisation of HBV transcripts in conjunction with enhanced reverse transcription of HBV pgRNA.

To precisely locate sites of m^6^A within HBV RNA, Imam and colleagues performed m^6^A-seq on uninfected and HBV-expressing hepatocytes and identified a single m^6^A peak at position A1907 in the HBV genome; however, they did not investigate any changes in m^6^A content of cellular transcripts [64]. The identified m^6^A site falls within a 3′ epsilon stem loop present in all HBV transcripts; though importantly, pgRNA possesses this m^6^A residue in both its 5′ and 3′ epsilon stem loop. To specifically determine the function of these m^6^A sites, the group created three mutants deficient in m^6^A at one or both of the pgRNA stem loops. By assessing protein levels, RNA half-life and viral DNA synthesis in core particles, Imam and colleagues demonstrated that 3′ m^6^A-mutant pgRNA is more stable than its wild type counterpart while 5′ m^6^A-deficient pgRNA undergoes less efficient reverse transcription. Furthermore, the pgRNA lacking m^6^A within both epsilon stem loops displays both of these phenotypes, recapitulating the effect seen for depletion of METTL3 and METTL14. The A1907C mutation leads to a base pair mismatch within the epsilon stem loop structure, thus m^6^A-deficiency could lead to structural alterations which might explain the effects on RNA stability and reverse transcription. Importantly however, the restoration of base pairing with a compensatory mutation could not reverse the decrease in protein expression and enhanced reverse transcription. As a result, Imam and colleagues provide strong evidence that methylation of A1907 in HBV RNA affects the virus life cycle through the modulation RNA stability and reverse transcription.

### 3.7 m^6^A in a plant virus

To date, very few studies have been conducted regarding the function of m^6^A in plants and very little is known about the m^6^A machinery in these organisms. Despite this, a recent study has expanded the field of viral epitranscriptomics by providing the first evidence of a functional role for m^6^A in the regulation of a plant virus. Martínez-Pérez et al. identified a member of the AlkB family of demethylases, ALKBH9B, among a yeast two-hybrid screen for interaction partners of the multifunctional alfalfa mosaic virus (AMV) coat protein (CP) in *Arabidopsis thaliana* [65]. After validating this interaction, Martínez-Pérez and colleagues compared the infective ability of AMV in an ALKBH9B-deficient *Arabidopsis* stock to wild type plants. They found that both vRNA and CP levels were significantly reduced in the mutants compared to wild type plants following AMV inoculation suggesting that viral infection is attenuated. Intriguingly, fluorescence microscopy studies showed that ALKBH9B overlaps perfectly with SGS3, a component of siRNA bodies, and DCP1, a decapping enzyme in P-bodies, suggesting ALKLBH9B m^6^A activity might be linked to mechanisms of mRNA silencing and decay that are conserved among eukaryotes.

To determine whether ALKBH9B is indeed an m^6^A eraser, Martínez-Pérez et al. assessed the ability of GST-purified ALKBH9B to remove m^6^A from a methylated RNA oligonucleotide [65]. The RNA substrate was almost entirely demethylated by ALKBH9B confirming the protein as an m^6^A eraser. Given the link between m^6^A and AMV infection, Martínez-Pérez and colleagues mapped the m^6^A landscape in AMV by MeRIP-seq and identified six putative m^6^A sites. Furthermore, the ALKBH9B-depleted *Arabidopsis* stock displayed a 35% increase in m^6^A levels. Together, these results suggest that the RNA hypermethylation in ALKBH9B-mutants impairs AMV infection and thus m^6^A negatively regulates the virus life cycle. Importantly however, depletion of ALKBH9B did not potentiate infection by cucumber mosaic virus (CMV), another *Arabidopsis* pathogen, despite its m^6^A-modified RNA genome suggesting ALKBH9B does not regulate CMV infection. Notably, a lack of interaction between ALKBH9B and CMV CP was identified which may explain this finding. Nevertheless, this recent publication provides exciting evidence that m^6^A plays a fundamental regulatory function in the life cycles of plant viruses, attesting to the ubiquitous nature of the modification in the field of virology.

## 4 Changes in the cellular m^6^A landscape during viral infection

While the m^6^A-methylation of viral genomes clearly plays a crucial role in regulating viral infection, changes in the host m^6^A landscape represent another mechanism for potentiating viral-host interactions. Five of the studies discussed previously chose to investigate this fascinating hypothesis by mapping the m^6^A methylome in both uninfected and virally-infected host cells. In each case, a set of uniquely or differentially methylated transcripts were identified and subjected to gene ontology (GO) analysis to discover enriched pathways by functional clustering. Lichinchi and colleagues identified 56 transcripts uniquely methylated under HIV-1 infection, for which the most represented category was viral gene expression [56]. Indeed, 19 of the protein products of these cellular mRNAs had been previously linked to HIV replication; a subset of which interact directly with HIV viral components and undertake mostly proviral functions. However, an identical investigation by Tirumuru et al. found that transcripts which were differentially methylated upon HIV-1 infection were functionally clustered in broader cellular pathways such as immunity, metabolism and development [57]. Similarly, during ZIKV infection, Lichinchi and colleagues also identified immune-related genes as those most enriched among cellular transcripts containing *de novo* m^6^A peaks [18]. In addition, Tan and others found that transcripts involved in pathways associated with oncogenesis and KSHV latency programmes were most enriched among those subjected to differential methylation during KSHV infection [61]. Conceivably however, differentially methylated transcripts involved in KSHV lytic replication were most abundant when comparing cells undergoing latent and lytic infection programmes. In contrast, Hesser and colleagues found a striking 25% decrease in m^6^A content upon cellular transcripts during KSHV induction, but could not find any notable enrichments in GO analysis of these downregulated transcripts implying that viral transcripts are prioritised for methylation during reactivation. Nevertheless, taken together, these results strongly evidence the re-organisation of the host m^6^A landscape in order to modulate viral infection; however, it remains unclear whether these observations are due to the cell mounting an antiviral response, viral subversion of host cell machinery or a combination of both of these phenomena.

To identify any changes in the cellular topology of m^6^A in response to viral infection, several of these studies compared the preference in m^6^A consensus site between uninfected and infected conditions. In HIV-1, Lichinchi and colleagues identified a 5% increase in m^6^A-methylated MGACK (A/C-GAC-G/U) motifs during infection [56]. However, Tirumuru and others found only a minor 0.2–0.8% and 0.2–0.4% in RRACH and GGACU motifs respectively [57]. In ZIKV infection, cellular m^6^A levels increased in 5′ UTRs and coding sequences while correspondingly decreasing in 3′ UTRs and exon junctions [18]. In addition, comparison of consensus site usage in ZIKV-infected and uninfected cells showed a loss in m^6^A from GAC sites and a gain at AAC sites. Finally, in KSHV-infected cells, changes in m^6^A distribution differed with cell type and in response to both latent and lytic replication programmes [61]. Although Tan and colleagues suggested GGAC as the most frequently utilised m^6^A consensus site under uninfected, latently-infected and lytically-infected conditions they did not compare changes in m^6^A motif usage. Importantly however, they identified that few of the differentially m^6^A-modified transcripts were likely to be sites of m^6^A_m_ suggesting a minimal effect for this related modification in initiating lytic replication. Together, these results suggest the cellular m^6^A landscape is dynamically regulated under viral infection, hinting towards the exciting possibility that the preferred consensus site of the m^6^A machinery may be altered in response to physiological conditions.

## 5 Concluding remarks and future perspectives

Although the m^6^A-modification of viral RNAs was first discovered several decades ago, the evidence gathered by just a small number of studies in the last two years has indicated that this ubiquitous and dynamic epitranscriptomic phenomenon likely plays widespread regulatory roles in a broad range of viruses. The development of precise methods for m^6^A mapping has established the nexus for systematically expanding our understanding of m^6^A function. Nevertheless, upgraded technologies permitting the direct sequencing of viral RNAs are now emerging which have the potential to abolish the difficulties and inaccuracies associated with current techniques [82]. Depletion of the host m^6^A machinery and abrogation of methylation at specific sites continue to be indispensable methods for determining m^6^A function in viral life cycles. However, the targeted methylation or demethylation of specific sites of modification, while technically challenging, would indisputably permit the elucidation of m^6^A function. Furthermore, the ability of m^6^A to positively regulate the replication of some viruses, while inhibiting others remains a key question that must now rise to the precipice for exploration. Similarly, it is unclear if changes in the host epitranscriptome, whether proviral or antiviral, play significant roles in modulating virus infection. The resolution of these outstanding questions will likely accelerate our understanding of m^6^A function in virus-host interactions and bring forth the coming enlightenment in viral epitranscriptomics.

## Transparency document

Transparency document. Image 1
